# SIDS and Infant Sleep Ecology

**DOI:** 10.1093/emph/eou023

**Published:** 2014-10-16

**Authors:** Helen L. Ball, Charlotte K. Russell

**Affiliations:** Parent-Infant Sleep Lab, Department of Anthropology, Durham University, Dawson Building, South Road, Durham, DH1 3LE, UK

## Sudden infant death syndrome

Sudden infant death syndrome (SIDS) is the designation given to the unexpected death of an infant that remains unexplained following post-mortem, death scene investigation and review of clinical history [1]*.* The search for mechanisms underlying such deaths has been largely unsuccessful; brainstem anomalies are thought to be involved. Although rare SIDS is the leading category of non-accidental deaths between 1 month and 1 year of age, annually affecting one in 3000 babies in the UK and one in 2000 in USA.

Key associations with SIDS are identified using retrospective studies of SIDS-cases and matched controls. Three key factors identified are: ‘Intrinsic infant vulnerability’ (premature birth, low birth weight, male gender and prenatal smoke exposure). Death during sleep (night or day) is typical, with peak prevalence during a ‘critical developmental phase’ at 2–3 months of age. Thirdly SIDS deaths involve ‘exposure to an external stressor’, a feature of sleep ecology imposing a physiological challenge on the infant. The confluence of an external stressor, a critical developmental period, and an intrinsically vulnerable infant are encapsulated in the Triple Risk Model for SIDS [2].

## Evolutionary perspectives

SIDS-deaths are a phenomenon of infant sleep in Western post-industrialized cultures, normally occurring while infants are alone. This pattern implicates aspects of infant sleep ecology prevalent in contemporary Euro-American societies that are incongruent with evolved infant physiology [3]. Immigrant groups who maintain their ‘traditional’ sleep ecology in ‘Western’ environments typically exhibit substantially lower SIDS rates than the host community [4].

Comparative evolutionary studies indicate that human infants are poorly neurologically developed at birth, requiring close physical contact for safety, physiological regulation and frequent feeding. Our species-specific sleep ecology involves close contact with a carer and frequent sleep arousals for the first 6 months of life [3].

Contemporary Euro-American sleep ecology encourages and values solitary and prolonged infant sleep from an early age, in combination with artificial milk feeding and the use of ‘sleep aids’ (such as pacifiers, white noise, swinging cradles and swaddling) to encourage infants to sleep deeply with minimal arousals. Inhibition of the arousal response is a characteristic of vulnerable babies and is enhanced by external stressor exposure [5].

## Future implications

Infant care strategies that promote early sleep independence fail to provide infants with arousal support during the critical physiological transitions that characterize the early months of life. Current SIDS prevention relies on identifying discrete sleep-related risks (such as prone position, soft surfaces) and advising parents to avoid these ‘modifiable risk-factors’ [2].

An evolutionary perspective suggests a more holistic view of infant sleep ecology is warranted. Clinicians can discourage infant-care trends that fail to support normal infant physiology (such as prolonged solitary sleep and early sleep consolidation), encourage parental proximity and responsive care, and educate parents about infant developmental needs.
